# Digital health literacy as mediator between language preference and telehealth use among Latinos in the United States

**DOI:** 10.1093/jamia/ocaf232

**Published:** 2026-01-13

**Authors:** Miguel Linares, Jorge A Rodriguez, Lauren E Wisk, Douglas S Bell, Arleen Brown, Alejandra Casillas

**Affiliations:** Division of General Internal Medicine and Health Services Research, David Geffen School of Medicine, University of California, Los Angeles, CA 90095, United States; Division of General Internal Medicine, Brigham and Women’s Hospital, Boston, MA 02115, United States; Harvard Medical School, Boston, MA 02115, United States; Division of General Internal Medicine and Health Services Research, David Geffen School of Medicine, University of California, Los Angeles, CA 90095, United States; Division of General Internal Medicine and Health Services Research, David Geffen School of Medicine, University of California, Los Angeles, CA 90095, United States; Division of General Internal Medicine and Health Services Research, David Geffen School of Medicine, University of California, Los Angeles, CA 90095, United States; Division of General Internal Medicine and Health Services Research, David Geffen School of Medicine, University of California, Los Angeles, CA 90095, United States

**Keywords:** digital health literacy, telehealth, Latinos, NELP

## Abstract

Using 2023-2024 U.S. National Health Interview Survey data, we found that digital health literacy (dHL) mediated nearly half of the difference in telehealth use between Latino adults with non-English and English language preference. These findings identify dHL as a modifiable mechanism linking linguistic and digital access barriers, underscoring the need for multilingual, inclusive, and equitable telehealth design.

## Introduction

Telehealth, delivered through video or audio encounters, rapidly expanded during the COVID-19 pandemic and remains above pre-pandemic levels.^1^ Yet patients with non-English language preference (NELP) use telehealth less often than those with English language preference (ELP) and report worse experiences.^2^^,^^3^ Structural barriers (eg, limited broadband or devices, lack of multilingual platforms, challenges integrating interpreter services) contribute to these differences and have additionally been linked to delays in care, suboptimal chronic disease monitoring, reduced quality of care, and ultimately worse health outcomes for patients with NELP.^2^^,^^4^

Digital health literacy (dHL), the ability to navigate, interpret, and communicate health information online, may also play a role.^5^ dHL not only enables access but also shapes trust, perceived usefulness, and the ability to act on information received through digital encounters, including patient portal use, secure messaging and video or telephone telehealth visits.^6^ Thus, understanding its role in telehealth disparities is critical for designing interventions that move beyond access toward meaningful engagement.

Latinos are the largest U.S. group with NELP. Compared to those with ELP, they use telehealth less often, and face greater linguistic and digital accessibility challenges. These include reliance on mobile-only internet or limited data plans rather than stable broadband, limited availability of high-quality Spanish translations or simplified interfaces within patient portals, and difficulties navigating multi-step authentication processes.^5^^,^^7^ Telehealth platforms also inconsistently support interpreter integration, contributing to worse communication experiences for patients with NELP.^2^^,^^5^ Prior studies have shown that Latinos with NELP have lower odds of possessing dHL compared to those with ELP.^7^ This cross-sectional study examines whether dHL mediates the association between language preference and telehealth use among Latinos in the U.S.

## Methods

We analyzed data from the 2023-2024 National Health Interview Survey (NHIS), a continuous nationally representative, cross-sectional survey of the civilian, non-institutionalized U.S. population. The NHIS is administered throughout the year using in-person and telephone interviews and employs a stratified, multistage probability sample of U.S. households. We focused on Latino adults 18 years of age or older with a usual source of care, defined as having “a place respondents usually go when they are sick or need health care.” Home language was categorized as ELP or NELP (defined by answering yes to the question *“Do you speak a language other than English at home?”*).

The primary outcome was self-reported telehealth use in the past 12 months. The mediator was a composite score from three NHIS measures of dHL skills, asked of individuals who reported having access to the internet: electronic communication with a clinician, reviewing test results digitally, and seeking health information online.^8^ Each item was dichotomized (yes/no) and summed to create a 0-3 composite score, with higher scores indicating higher levels of digital skills.

Covariates included age, sex, education, marital status, federal poverty level, insurance, nativity, employment status, geography and survey year. These variables were chosen a priori based on prior research identifying sociodemographic and structural factors associated with both telehealth use and language preference.^9^^,^^10^ Missing data on key variables were handled via complete case analysis. Weighted missingness exceeded 5% for language preference (12.2%) and nativity (5.5%); so, we estimated models both with and without treating missingness as a separate category. Results were substantively similar, so models excluding missing categories are presented.

We used survey-weighted Poisson regression with robust variance estimation to model prevalence ratios (PRs). To test mediation, we evaluated how the coefficient for language preference changed after adding the composite dHL score. As a sensitivity analysis, we repeated the mediation model treating each of the three dHL components as separate covariates rather than a composite score to assess the robustness of the findings. The proportion mediated was calculated as the ratio of the indirect effect to the total effect. The Sobel test evaluated the statistical significance of the indirect effect.^11^^,^^12^ Analyses were conducted using Stata 17 (StataCorp LLC). Survey design variables were applied according to NHIS guidance, with rescaled weights for pooled years to ensure that estimates represented the average annual U.S. Latino population.

Since this study used publicly available data, the UCLA IRB granted an exemption and waived informed consent.

## Results

The pooled analytic sample included 5,851 adults, representing 29.2 million U.S. Latinos per year (Table 1). Of these, 75.8% reported NELP, and 24.2% reported a telehealth visit in the prior year, with lower use among those with NELP (22.3%) compared with those with ELP (30.4%) (*P <.001*). Compared with ELP, adults with NELP were more often female, had lower education and income, were more likely to be uninsured or covered by Medicaid, and less likely to be employed.

**Table 1.** Weighted baseline characteristics of participants by language preference: 2023-2024 National Health Interview Survey.

	Participants, No. (%) (*n* = 5851, unweighted) (*N* = 29 222 482 weighted)				
Characteristic	NELP		ELP		*P* value
	(*N* = 22 138 952)		(*N* = 7 083 530)		
**Age group, *y***					
18-39	10 028 945	45.3	3 847 773	54.3	<.001
40-49	4 447 716	20.1	1 161 699	16.4	
50-64	4 963 553	22.4	1 326 037	18.7	
≥65	2 696 524	12.2	748 021	10.6	
**Sex**					
Male	9 639 300	43.5	3 468 096	49.0	.002
Female	12 499 653	56.5	3 615 434	51.0	
**Education level**					
Less than high school	6 271 965	28.3	795 480	11.2	<.001
High school	6 376 018	28.8	1 919 637	27.1	
Some college	5 415 188	24.5	2 409 817	34.0	
College degree or higher	4 075 781	18.4	1 958 596	27.7	
**Employment**					
Employed	14 903 943	67.3	5 060 474	71.4	.01
Unemployed^a^	7 235 010	32.7	2 023 056	28.6	
**Geography**					
Nonmetro	1 186 648	5.4	430 749	6.1	.19
Medium/small metro	5 364 268	24.2	1 878 552	26.5	
Suburban	4 615 972	20.9	1 578 210	22.3	
Large central metro	10 972 065	49.6	3 196 089	45.1	
**Marital status**					
Unmarried	10 976 493	49.6	4 013 528	56.7	<.001
Married	11 162 460	50.4	3 070 002	43.3	
**Annual household income percentage of federal poverty level**					
<100	3 491 313	15.8	661 673	9.3	<.001
100-199	6 285 249	28.4	1 200 658	17.0	
≥200	12 362 391	55.8	5 221 270	73.7	
**Insurance**					
Any private insurance	10 527 072	47.6	4 589 419	64.8	<.001
Medicaid/dual eligible	5 479 391	24.8	1 178 699	16.6	
Medicare only	1 414 015	6.4	427 845	6.0	
Uninsured	3 914 167	17.7	487 701	6.9	
Other	804 972	3.6	400 219	5.7	
**Telehealth use in previous 12 months**					
Yes	4 941 414	22.3	2 149 851	30.4	<.001

a The unemployed included those not employed or not in the labor market at the time of the survey.

**Figure 1.**
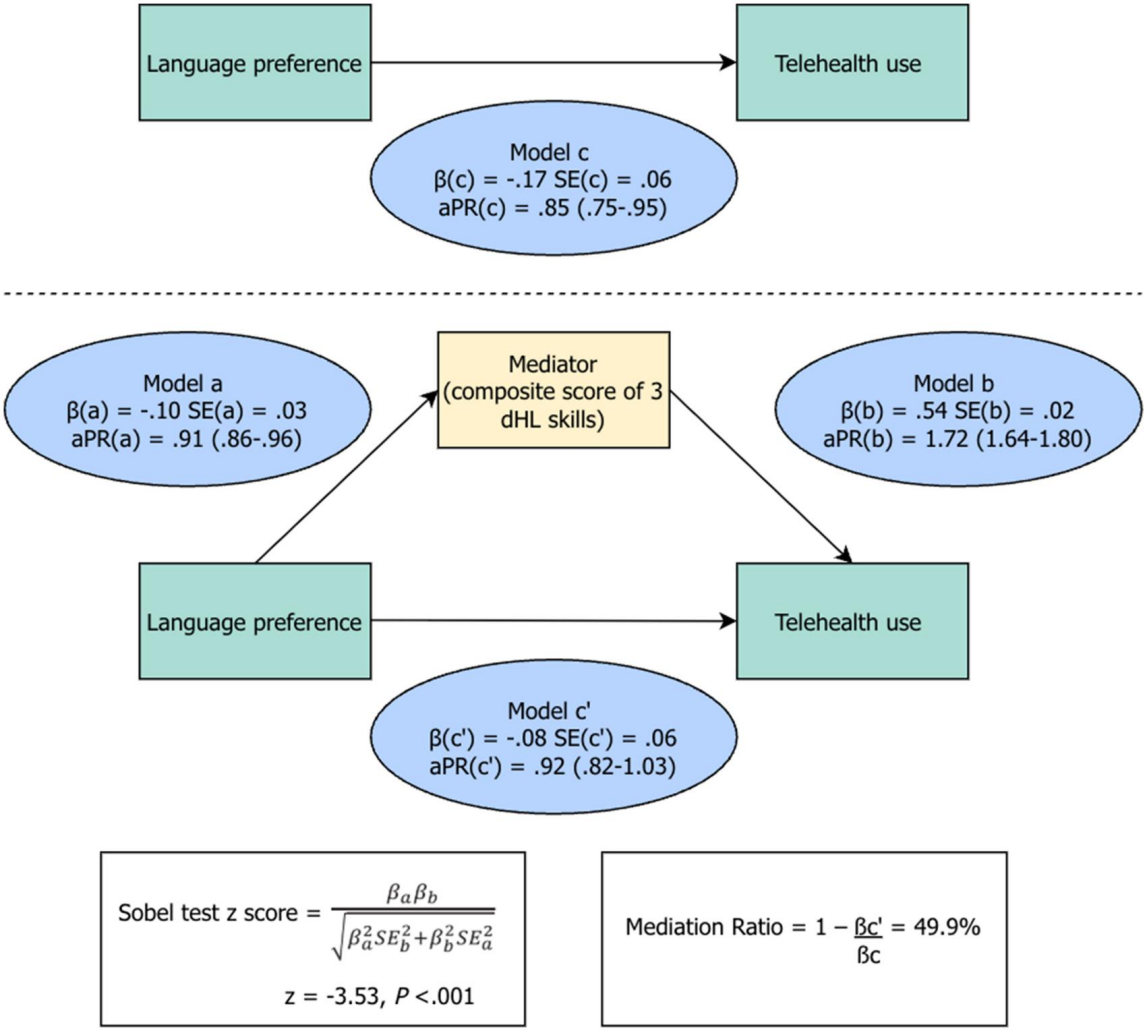
Conceptual mediation pathway between language preference and telehealth use via Digital Health Literacy (dHL).^a^ **Legend**: Model c shows the total association between language preference and telehealth use. Model a assesses the association between language preference and the mediator (composite dHL score). Model b estimates the association between the mediator and telehealth use, adjusted for language preference. Model c′ represents the direct association between language preference and telehealth use after accounting for dHL. The indirect (mediated) effect was tested using the Sobel *z* test. ^a^ Path coefficients (βs) are from survey-weighted Poisson regression models with robust variance estimation. * aPR = adjusted Prevalence Ratios.

In the adjusted model (Figure 1, model c), Latino adults with NELP were less likely than those with ELP to report telehealth use (PR = 0.85; 95% CI, 0.75-0.95; *P = .007*). Patterns consistent with mediation were observed: NELP additionally predicted lower dHL (Figure 1, model a), and higher dHL (the proposed mediator) was associated with greater telehealth use while adjusting for language preference (Figure 1, model b); when dHL was added to model c, the association between NELP and telehealth use attenuated towards the null and was no longer statistically significant (PR = 0.92; 95% CI, 0.82-1.03; *P = .16*) (Figure 1, model c’). Mediation analysis indicated that dHL explained 49.9% of the language preference-telehealth association (Sobel *z* = −3.53; *P <.001*) (Figure 1). In sensitivity analysis of model c’, replacing the composite dHL score with its individual components, results were similar (PR = 0.93; 95% CI, 0.83-1.04; *P = .23*), supporting the robustness of the mediation findings.

## Discussion

This study’s findings suggest that dHL mediates disparities in telehealth use among Latino adults with NELP, with half of the disparity explained by dHL skills. This finding highlights that language-related inequities in telehealth are intertwined with broader digital divides, highlighting dHL as an actionable mechanism linking linguistic and technological access barriers.

In this national sample, Latino adults with NELP also experienced substantial socio-economic disadvantages, including lower educational attainment, lower income, higher rates of being uninsured or Medicaid-insured, and lower employment, relative to those with ELP. These structural factors have been previously described for this population^13–15^ and are tightly linked to the development of dHL as limited educational and economic resources can restrict both material access to digital tools but also opportunities to build the skills required to use them effectively.^7^^,^^10^ Consequently, dHL has been described to function as a “super determinant” of health that both reflects and amplifies these broader social inequalities.^6^^,^^8^

In the era of expanding telehealth and artificial intelligence, tools that promote equitable dHL are essential, and the findings from this study highlight the importance of strengthening dHL skills. To move beyond general accessibility, interventions must target specific dHL skills, including those evaluated in this study.^16^^,^^17^ For electronic communication, prior work shows that Spanish speakers often prefer modalities that align with existing user behaviors, such as text messaging, suggesting that portal-based communication tools may be insufficient for populations with NELP.^18^^,^^19^ To support patient review of test results, health systems should incorporate upstream digital aids, such as in-portal video explanations or automated translations, that help patients interpret clinical data in their preferred language through simple, one-click processes.^4^^,^^5^ Enhancing online information seeking requires addressing the scarcity of trusted non-English digital content by developing curated, culturally responsive information and offering community-based training to support evaluation of online health information.^6^^,^^8^ Complementary strategies, including device access, multilingual digital education programs, simplified patient portal interfaces, and culturally responsive design, can further strengthen these domain-specific skills and promote more meaningful digital engagement.^17^ Notwithstanding, interventions aimed at improving dHL skills may reduce, but not fully eliminate, language-related disparities. Meaningful progress toward digital and multilingual health equity will require addressing upstream determinants, such as infrastructure, literacy, and linguistic accessibility through coordinated efforts among policymakers, health systems, and technology.^4^^,^^20^

This study has limitations, and several contextual considerations are important. First, the NHIS excludes institutionalized populations and relies on self-reported telehealth use and digital skills, which may introduce measurement error as well as recall or reporting bias. Second, dHL was assessed using three NHIS measures of digital skills that capture core elements of digital engagement but do not encompass the full complexity of dHL. Third, since this is a cross-sectional study, it cannot confirm the directionality of associations between dHL and telehealth use; engaging in telehealth may also improve an individual’s dHL over time. Notwithstanding these limitations, the generalizability of our findings is strengthened by the use of a large, nationally representative survey that reflects the heterogeneity of the U.S. Latino population across regions, nativity, socioeconomic status, and digital access contexts. To better evaluate system-level interventions and advance equitable digital engagement across language groups, future work should leverage longitudinal EHR data and incorporate community engagement and participatory design approaches with Latino patients to ensure that digital tools reflect their lived experiences and linguistic needs.

## Data Availability

The data underlying this article are publicly available from the National Health Interview Survey (NHIS) and can be accessed through the CDC’s website at https://www.cdc.gov/nchs/nhis/.
